# Antiretroviral Use for HIV Prevention During Pregnancy: The Need to Strengthen Regulatory and Surveillance Systems in Africa

**DOI:** 10.1007/s40264-024-01494-6

**Published:** 2025-01-26

**Authors:** Robin Schaefer, L. Donaldson, A. Chigome, M. Escudeiro dos Santos, S. Lamprianou, N. Ndembi, J. I. Nwokike, P. Nyambayo, V. Palmi, F. Renaud, M. Gonzalez Tome, V. Miller

**Affiliations:** 1 Forum for Collaborative Research, University of California, Berkeley, Washington, DC USA; 2 South African Health Products Regulatory Authority, Pretoria, South Africa; 3 Vaccines and Therapies for Infectious Diseases, Human Medicines Division, European Medicines Agency, Amsterdam, The Netherlands; 4 Pharmacovigilance team, Department of Regulation and Prequalification, World Health Organization, Geneva, Switzerland; 5 Africa Centres for Disease Control and Prevention, Addis Ababa, Ethiopia; 6 United States Agency for International Development (USAID)’s Promoting the Quality of Medicines Plus (PQM+) Program, United States Pharmacopeia, Rockville, MD USA; 7 Medicines Control Authority of Zimbabwe, Harare, Zimbabwe; 8 International Affairs, European Medicines Agency, Amsterdam, The Netherlands; 9 Global HIV, Hepatitis, and STIs Programmes, World Health Organization, Geneva, Switzerland

## Abstract

HIV-prevention efforts focusing on women of child-bearing potential are needed to end the HIV epidemic in the African region. The use of antiretroviral drugs as pre-exposure prophylaxis (PrEP) is a critical HIV prevention tool. However, safety data on new antiretrovirals during pregnancy are often limited because pregnant people are excluded from drug development studies. Calls from communities, healthcare professionals, and regulators to improve the information supporting decision-making around the use of medical products during pregnancy have been increasing. Post-marketing safety surveillance is an essential tool for detecting adverse outcomes and evaluating real-world, longer-term effects of drugs. Detecting and evaluating uncommon pregnancy outcomes requires large sample sizes, highlighting the benefits of and need for safety surveillance. Surveillance systems vary widely across Africa, and the need for enhanced surveillance of PrEP use during pregnancy highlights the limitations of current regulatory and surveillance systems. Challenges include weak regulation and insufficient resources. Pooling of resources and regulatory harmonization could address resource challenges. The African Medicines Agency, as a specialized agency of the African Union, has the potential to improve African medical product regulation, including post-marketing safety surveillance. This can strengthen regulation and ensure that market authorization holders meet their responsibility to invest in post-marketing surveillance systems, such as pregnancy registries. At the same time, independent post-marketing studies are needed to ensure generation of essential safety data. The Forum for Collaborative Research has initiated a project to facilitate interactions between regulators in Africa, the USA, and Europe, as well as other stakeholders, and to work toward consensus on safety data generation from PrEP during pregnancy before and after marketing authorization.

## Key Points

**Table:** 

Antiretroviral drugs are a critical HIV-prevention tool to end the HIV epidemic in the African region, but safety data on use during pregnancy are often limited.
Post-marketing safety surveillance is required to evaluate medical product safety during pregnancy, but surveillance systems in many African countries face challenges such as weak regulation and insufficient resources.
The African Medicines Agency has the potential to improve all aspects of African medical product regulation, and it is critical that this includes strengthening post-marketing surveillance.

## HIV Prevention During Pregnancy and Missing Inclusion, Safety, and Efficacy Information of Prevention Products

Adolescent girls and women are at substantial risk of HIV acquisition in many African countries [1, 2], notably in eastern and southern Africa, where 260,000 females aged ≥15 years acquired HIV in 2023, including 120,000 young women aged 15–24 years [3]. The incidence of HIV infection can be particularly high during pregnancy and the postpartum period [4, 5]. This is due to a combination of biological factors associated with pregnancy and the postpartum period [5] as well as psychosocial (e.g., changes in risk perception), behavioral (e.g., decreased condom use), and socio-cultural (e.g., beliefs around condom use during pregnancy) factors [6, 7]. Moreover, HIV acquisition during pregnancy and lactation contributes to vertical transmission of HIV [8], accounting for about 23,700 vertical HIV infections—about one-quarter of all vertical infections—in the World Health Organization (WHO) African region in 2023 [3]. Therefore, to end the HIV epidemic and eliminate vertical transmission of HIV, prevention efforts focusing on women^1^ of child-bearing potential and ensuring access to HIV prevention options during pregnancy and lactation are critical, together with early diagnosis and initiation of treatment as well as infant prophylaxis [9].

The use of antiretroviral drugs (ARVs) as pre-exposure prophylaxis (PrEP) is a critical tool in HIV prevention. There has been a considerable increase in PrEP use, with about 3.5 million PrEP users globally in 2023, but significant further growth is needed to meet the global target of 21.2 million users by 2025, with 4.5 million users in the African region alone [3]. The WHO recommends offering several PrEP products to all people at substantial risk of HIV acquisition. This includes tenofovir disoproxil fumarate (TDF)-containing oral PrEP, the dapivirine vaginal ring (DVR), and long-acting injectable cabotegravir (CAB-LA). Additional PrEP products are under development [10], including a 6-monthly injection with lenacapavir (LEN), which has recently been found to be efficacious and safe in phase III clinical trials [11, 12]. The WHO has also released guidelines to increase access to and use of post-exposure prophylaxis (PEP) – the use of ARVs to prevent HIV acquisition after exposure—through community-based delivery and task sharing [13]. Nevertheless, safety data for new ARVs during pregnancy (for PrEP, PEP, or HIV treatment) are often limited, creating challenges for the implementation of HIV prevention for this population.

Preclinical studies provide important information on the safety of new drugs, including possible teratogenicity, but pregnancy has commonly been an exclusion criterion in clinical studies during drug development. When drugs receive regulatory approval, labels tend to include limited information for use during pregnancy and lactation [14]. The lack of clinical data on the use of medical products during pregnancy and lactation can limit access and lead to precautionary measures to avoid or discontinue use. It may also result in ongoing use of potentially unsafe products. Even where approved labels may not explicitly restrict use during pregnancy, national guidelines may recommend against such use. For HIV treatment and prevention drugs, there are commonly delays of multiple years between the initial regulatory approval and pregnancy safety data becoming available [15, 16]. This is partly due to regulatory frameworks focusing historically on risks for those exposed to medical products and liability concerns among pharmaceutical companies [17], resulting in a lack of robust and reliable pregnancy-related data and a general exclusion of women in drug development.

Oral PrEP was first approved by the US Food and Drug Administration (FDA) in 2012. In 2015, the WHO recommended oral PrEP to be offered to all people at substantial risk of HIV acquisition and – in 2017 – provided guidance for use in pregnancy and lactation [18]. However, a lack of evidence on safety during pregnancy led to restrictive national regulatory approvals, and – even though there is now mounting evidence on the safety of TDF during pregnancy [19, 20], including through the international Antiretroviral Pregnancy Registry [19, 21] – implementation in this population remains limited [22]. By the end of 2023, DVR had been approved in 11 African countries with a significant burden of HIV [23]. However, DVR use during pregnancy has been restricted in some countries. For instance, in South Africa, the regulatory approval of the DVR notes a lack of pregnancy-specific data but highlights the need to balance benefits with harms, yet national guidance on the implementation of the DVR explicitly recommends against use during pregnancy [24]. Phase III clinical trials on the DVR enrolled only non-pregnant participants and required the use of contraception, although limited data suggested no significant difference in adverse pregnancy or infant outcomes between users of the DVR and placebo [25]. Subsequent open-label studies on DVR use during the second and third trimesters found adverse pregnancy outcomes to be uncommon and similar to rates observed in surrounding study communities [26, 27]. CAB-LA is an integrase strand transfer inhibitor, and preclinical studies suggest potential teratogenicity of integrase strand transfer inhibitors [28]. In 2018, a relatively small amount of data on the use of the integrase strand transfer inhibitor dolutegravir during pregnancy in Botswana raised the potential of an increased risk of neural tube defects [29]. This safety signal was refuted as corrected data from an increased number of exposures showed similar rates of birth defects between pregnant women exposed to dolutegravir and other antiretroviral drugs [30, 31]. These concerns led to a requirement for long-acting reversible contraception during clinical trials of CAB-LA, which limited the generation of clinical data on safety during pregnancy. Regulatory approvals to date highlight considerations of benefits and risks of use during pregnancy, and more data are becoming available as the contraceptive requirement has been removed during the open-label extension of the trial [32]. Planned and ongoing studies and implementation science projects that offer different PrEP products include pregnant participants [33]. Some of these studies will evaluate outcomes among exposed infants [34]. CAB-LA is also monitored through the Antiretroviral Pregnancy Registry [21], which will generate further evidence on the safety of CAB-LA during pregnancy.

In recent years, there have been increasing efforts toward the inclusion of pregnant participants in HIV clinical trials [35–37], and regulatory agencies have emphasized the need to generate non-clinical and clinical data on safety during pregnancy [38, 39]. PURPOSE 1, a large HIV-prevention trial of LEN among cisgender women in Uganda and South Africa, allowed participants to continue receiving study drugs after becoming pregnant [11]. Such inclusion of pregnant women in clinical trials has the potential to generate important information on the safety of PrEP during pregnancy, when maternal, pregnant, and infant outcomes are systematically recorded. However, detecting uncommon outcomes, such as rare pregnancy outcomes that occur at rates of fewer than 1 per 1000 exposures, and evaluating associations with drug exposure require large sample sizes that are unlikely to be provided by clinical trials alone [40]. This highlights the need for surveillance after product authorization to detect less common and longer-term effects, including longer-term effects on infants. Safety surveillance, therefore, has a critical role in ensuring access to safe medical products for pregnant women and promoting confidence among users that products are safe. The WHO encourages countries to implement surveillance for ARV use during pregnancy, including PrEP [18, 41], and has produced guidance for optimizing surveillance of ARV safety in pregnancy and harmonizing pregnancy safety data [42, 43].

In this article, we discuss surveillance approaches for PrEP during pregnancy, how the need for surveillance during pregnancy highlights limitations in current surveillance and regulatory systems in the African region, and approaches to strengthen these. We also introduce a project that aims to generate consensus among diverse stakeholders on the generation of safety data for PrEP during pregnancy before and after marketing authorization.

## Surveillance: A Core Regulatory Function

Safety monitoring of medical products is a core function of regulatory agencies. Historically, the focus of drug regulatory systems has been on pre-market evidence generation and marketing authorization of pharmaceutical products, with less attention devoted to post-marketing safety monitoring [44]. Drug approvals are based on a specific methodology involving clinical trials that randomize exposure, which is limited by, among others, their sample size, participant characteristics, and study duration. Therefore, post-marketing surveillance is necessary to understand a drug’s real-world, population-specific, and long-term effectiveness and safety [45]. Pharmacovigilance is the cornerstone of post-marketing safety surveillance. It is the science of the prevention, detection, investigation, and causality assessment of adverse events. To evaluate causality and the use of a medical product in a population and its public health risks and benefits, pharmacoepidemiologic methods can be applied, including case–control and cohort studies. These allow for comparisons between those exposed to a drug and those not. In post-marketing safety surveillance, etiological evaluations of associations between exposure to a drug and outcomes to determine causality are particularly challenging. Surveillance priorities may be informed by guided in vitro, animal, or human data submitted during regulatory filings or emerging after regulatory approval.

With methods initially developed in the 1960s [46], passive reporting of individual suspected adverse events, referred to as individual case safety reports (ICSRs), continue to be central to post-marketing safety surveillance. These are usually reported by healthcare professionals to national regulatory authorities (NRAs). ICSRs are the primary means of signal management [47]. In pharmacovigilance, a safety signal is information on a new or known adverse event potentially associated with an active substance or a medical product. Signal management refers to the set of activities performed to determine whether there are new risks or whether known risks have changed. These activities include the detection, validation, confirmation, analysis, prioritization, and assessment of signals – based on aggregated data from surveillance systems and the scientific literature – as well as any related recommendations, decisions, communications, and tracking. Pharmacoepidemiologic methods can be used to etiologically evaluate any newly identified risks or changes in known risks and quantifying the public health threat. Marketing authorization holders (MAHs) are legally obliged to report serious adverse reactions to their products to NRAs.

Post-authorization surveillance can generate larger sample sizes that can aid in evaluating less common and longer-term pregnancy outcomes [41]. Passive surveillance through ICSRs is common. However, it relies on voluntary reporting by healthcare providers and patients, which may lead to underreporting and biased data. Other exposures may have occurred between the drug exposure and detection of pregnancy or infant outcomes, confounding spontaneous reporting as a tool for signal detection of pregnancy-related events. Additional challenges with this approach include the lack of a reliable denominator and comparison group [48]. Finally, lack of standardization of exposure and outcome measures may impact data quality. For instance, gestational dating is often challenging but necessary to accurately ascertain exposure timing. The WHO Programme for International Drug Monitoring has a global database of ICSRs, allowing for pooling of data, but contributions are mainly from high-income countries [49]. In contrast, active surveillance systems do not rely on voluntary safety reporting. Approaches include sentinel site-based surveillance, in which data on pregnancy exposure and outcomes are actively collected on an often larger cohort of women; case–control studies, involving matched control groups of individuals without the outcome of interest; and cohort studies that prospectively or retrospectively collect data through pregnancy and beyond. Active surveillance systems address some of the limitations of passive surveillance and exist in high-income countries, mainly in North America and Europe [50], but are more limited in Africa [51]. For pregnancy surveillance, the use of sentinel sites, such as collecting data in high-volume delivery facilities, has been found to be appropriate [41]. Examples of active surveillance systems exist in Africa, such as the Tsepamo study in Botswana [29], the Western Cape Pregnancy Exposure Registry [52], and the UBOMI BUHLE project in South Africa [48]. There are also a range of pregnancy exposure registries in the African region [53] as well as the global Antiretroviral Pregnancy Registry that collects information on PrEP exposures [21].

## Challenges Faced by African Surveillance Systems and Regulatory Systems Strengthening

African regulatory authorities are at different maturity levels in the area of post-marketing safety surveillance. Surveillance systems vary widely in the region and face significant challenges, such as weak regulation and insufficient resources. Most African countries do not have legal provisions for requesting the MAH of a product to submit responses to local safety concerns generated from spontaneous reporting, and African regulators rarely request post-marketing studies [54, 55]. In the USA, the FDA Amendments Act of 2007 allows the FDA to mandate post-marketing studies [56]. Similarly, the EU enacted pharmacovigilance regulation that empowers regulators to request post-marketing studies [57].

The need for enhanced surveillance of the use of PrEP during pregnancy highlights the limitations of current regulatory and surveillance systems in Africa. Lack of financial and technical resources and limited collaboration are major limitations, and reliance on funding from external parties makes long-term planning challenging [55, 58]. The pooling of resources and regulatory reliance and harmonization are key to addressing the resource challenges [54]. Reliance approaches are commonly used for the authorization of medical products and should also be used for post-authorization activities, given the significant resources required for post-marketing surveillance over the course of a product’s life cycle [59]. The African Medicines Agency (AMA), as a specialized agency of the African Union, was established to enhance states and regional economic communities in medical product regulation [60]. It aims to coordinate regulatory systems and promote cooperation and harmonization between national and regional agencies and has the potential to improve all aspects of African medical product regulation [61]. Surveillance is one of the core functions of the AMA; it aims to coordinate and facilitate information exchange and harmonize efforts [60]. The AMA is intended not to replace but to complement and coordinate NRAs and regional initiatives.

Harmonization of systems can also strengthen regulation and ensure that MAHs meet their responsibility to couple the introduction of new medical products with investments in post-marketing surveillance systems, such as pregnancy registries. At the same time, independent post-marketing studies are needed to ensure adequate and timely generation of essential safety data. Several implementation studies that will offer multiple PrEP options, including CAB-LA, and enroll pregnant participants are planned in the African region [33]. Coordination across projects, including, where possible, harmonization of data collection and engagement with regulatory agencies, is critical to ensure that appropriate data for informing national policy and regulatory decision-making are generated. Simultaneously, pragmatic approaches to surveillance are needed [62]. This could include strengthening the reporting of adverse events by product users, including web-based systems that facilitate reporting by individuals, for instance by using their cell phones, or establishing contact help centers. For example, the African Union’s Smart Safety Surveillance program promotes the use of the mobile Med Safety App to facilitate reporting of suspected adverse events by healthcare professionals and members of the public, and is building AfriVigilance, a common African safety database [54]. Training and mentoring programs dedicated to healthcare professionals and local regulators can strengthen systems and improve outcomes [63]. The AMA aims to focus on developing regulatory science capacity, including in surveillance [60]. This is achieved through technical committees and regional centers of regulatory excellence that provide support and strengthen national agencies in areas where capacity may be limited. This strengthening of surveillance systems, including through active surveillance, can increase a national regulatory authority’s maturity level as per the WHO Global Benchmarking Tool [64].

## A Working Group to Support Interactions Between Regulators and Other Stakeholders

Calls from stakeholders, including communities, healthcare professionals, and regulators, to improve the information supporting decision-making for the use of medical products during pregnancy and lactation have been increasing. As the AMA will accelerate regulatory harmonization in Africa and aims to strengthen regulatory systems, the focus must not be limited to pre-market evidence generation and marketing authorization. Post-marketing safety surveillance is critical to ensure the safety of medical products, such as PrEP, and the AMA will be key in promoting collaboration among African countries and regions to streamline decision-making and standardize reporting tools. All aspects of safety surveillance need to be strengthened, from spontaneous reporting, case definition, and adoption of international ICSR data standards to active surveillance approaches, such as the use of sentinel sites and cohort event monitoring, and evaluation and mitigation of potential risks. Training to support pregnancy registries is vital [65].

The Forum for Collaborative Research (the Forum) [66], based at the University of California, Berkeley, USA, has initiated a project that aims to support existing and emerging mechanisms of exchange between regulatory agencies through the African Union and facilitate interactions between regulators in Africa, the USA, and Europe, as well as other stakeholders, on the use of biomedical HIV prevention in diverse populations. The Forum receives unrestricted grants from the pharmaceutical industry, but these are not specifically linked to this project, which is funded by the Bill & Melinda Gates Foundation. Building on decades of experience by the Forum in facilitating multistakeholder dialogue, including previous projects on safety monitoring for HIV treatment [67–69], the Forum aims to provide a neutral and independent space where all stakeholders have an equal voice in discussion. A working group has been established with representatives from regulatory agencies, normative bodies, research, industry, and communities. The group aims to initiate vital discussions and work toward consensus on the generation of safety data for PrEP during pregnancy before and after product marketing authorization. The group will meet every 4–6 weeks and identify and discuss safety surveillance data harmonization, outcomes of interests, and implementation of tools and procedures, among others. Lessons learned from post-marketing surveillance efforts in HIV treatment and prevention (such as for dolutegravir [70]) and beyond will be reviewed. Although the group will specifically focus on the use of PrEP, discussions will be anchored on the need to strengthen health systems more broadly, and disease- and product-specific surveillance systems may be leveraged as new products become available and are extended beyond their initial focus [53]. The working group includes expert representation from the pharmaceutical industry as MAHs have a responsibility to contribute to post-marketing surveillance efforts, and involvement of industry partners is critical for data harmonization and collaboration. However, the working group aims to benefit the field broadly, not a specific stakeholder, and does not aim to promote one particular medical product. The group aims to disseminate its deliberations and recommendations, formed through consensus, in future articles.

## Conclusions

HIV prevention efforts focusing on women of child-bearing potential and ensuring access to biomedical prevention options during pregnancy and lactation are critical to ending the HIV epidemic. However, despite recent advances concerning the inclusion of pregnant people in clinical trials, safety data on new ARVs during pregnancy are often limited at the time of product authorization, highlighting the need to accelerate and enhance post-approval safety surveillance. Challenges faced by regulatory and surveillance systems in the African region include lack of financial and technical resources and limited collaboration. Regulatory harmonization and reliance under the AMA have the potential to improve major aspects of medical product regulation, including safety surveillance. To facilitate interactions between regulatory agencies and other stakeholders on the use of biomedical HIV prevention, the Forum has formed a working group to generate consensus on safety data collection for PrEP during pregnancy. Ultimately, the group aims to ensure that safe and effective prevention options are more rapidly accessible for those who could benefit from them, including those who are pregnant, and post-marketing safety surveillance is central to these efforts.

## Footnotes

1 The terms ‘woman’ and ‘mother’ are intended to be inclusive of all those who identify as women and/or who give birth. While most people who are giving or can give birth are cisgender women (who were born and identify as female), our vision is also inclusive of the experiences of transgender men and other gender-diverse people who have the reproductive capacity to give birth.
